# Impact of climate change on the tick-host-pathogen complex: distribution patterns, disease incidence, and host infestation

**DOI:** 10.1590/S1984-29612025062

**Published:** 2025-10-27

**Authors:** Saeed Mohammed Nasser Alasmari, Chi-Wen Tu, Mehran Khan, Bushra Javed, Iram Liaqat, Sher Bahadar, Sarah Abdulaziz Altwaim, Chien-Chin Chen, Itabajara da Silva Vaz, Abid Ali

**Affiliations:** 1 Najran University, Faculty of Science and Arts, Najran, Saudi Arabia; 2 Ditmanson Medical Foundation Chia-Yi Christian Hospital, Department of Surgery, Chia-Yi City, Taiwan; 3 Abdul Wali Khan University Mardan, Department of Zoology, Mardan, Khyber Pakhtunkhwa, Pakistan; 4 Government College University, Department of Zoology, Lahore, Punjab, Pakistan; 5 Abdul Wali Khan University Mardan, College of Veterinary Science, Mardan, Khyber Pakhtunkhwa, Pakistan; 6 King Abdulaziz University, Faculty of Medicine, Department of Clinical Microbiology and Immunology, Jeddah, Saudi Arabia; 7 King Abdulaziz University, King Fahd Medical Research Center, Jeddah, Saudi Arabia; 8 Ditmanson Medical Foundation Chia-Yi Christian Hospital, Department of Pathology, Chiayi, Taiwan; 9 Chia Nan University of Pharmacy and Science, Department of Cosmetic Science, Tainan, Taiwan; 10 National Chung Hsing University, Doctoral Program in Translational Medicine, Taichung Taiwan; 11 National Cheng Kung University, College of Bioscience and Biotechnology, Tainan, Taiwan; 12 Universidade Federal do Rio Grande do Sul – UFRGS, Centro de Biotecnologia, Porto Alegre, RS, Brasil; 13 Universidade Federal do Rio Grande do Sul – UFRGS, Faculdade de Veterinária, Porto Alegre, RS, Brasil; 14 Instituto Nacional de Ciência e Tecnologia em Entomologia Molecular – INCT-EM, Rio de Janeiro, RJ, Brasil

**Keywords:** Ticks, climate change, hosts, pathogens, tick-borne diseases, Carrapato, mudança climática, hospedeiro, patógeno, doenças transmitidas por carrapatos

## Abstract

Ticks, being ectothermic, are highly sensitive to climate variables, such as temperature, humidity, and precipitation. Over the past century, fossil fuel use has altered the climate and significantly affected the tick-host-pathogen system. These changes influence tick lifecycles, behavior, vector competency, host dynamics, and pathogen transmission. Consequently, tick-borne diseases (TBDs) have experienced shifts in their geographical range, incidence, and host preferences, particularly in the Northern Hemisphere. While climate change drives the emergence of vector-borne diseases, key aspects, such as tick infestations on alternative hosts, remain understudied. However, some studies have highlighted the establishment of ticks and tick-borne pathogens (TTBPs) in previously unaffected areas of Europe and North America, dispersed through hosts migration, including birds. Understanding these changes is crucial for mitigating the risks to public health, livestock, and wildlife. This review examined geographical spread of TTBPs, TBD incidence, and alternative host infestations to identify challenges and opportunities for disease control. Since TBD epidemiology is also shaped by other anthropogenic factors, isolating climatic impacts is difficult. Multidisciplinary approaches that combine ecological modeling, molecular research, and surveillance are essential for clarifying climate-driven trends and improving TBD management.

## Resumo

Carrapatos, por serem ectotérmicos, são altamente sensíveis a variáveis climáticas como temperatura, umidade e precipitação. Ao longo do último século, o uso de combustíveis fósseis alterou o clima, impactando significativamente o sistema carrapato-hospedeiro-patógeno. Essas mudanças influenciam o ciclo de vida, a competência vetorial dos carrapatos, a dinâmica dos hospedeiros e a transmissão de patógenos. Consequentemente, as doenças transmitidas por carrapatos (TBDs) apresentam mudanças em sua distribuição geográfica, incidência e preferência por hospedeiros. Embora as mudanças climáticas impulsionem o surgimento de doenças vetoriais, aspectos, como infestações em hospedeiros alternativos, ainda são pouco estudados. Pesquisas destacam a expansão de carrapatos e patógenos por eles transmitidos (TTBPs) em áreas anteriormente não afetadas, dispersos pela migração de hospedeiros, incluindo aves. Compreender essas mudanças é crucial para mitigar os riscos à saúde pública, à pecuária e à vida selvagem. Esta revisão examina a expansão geográfica dos TTBPs, a incidência de TBDs e as infestações em hospedeiros alternativos. Como a epidemiologia das TBDs também é influenciada por outros fatores antropogênicos, isolar os impactos climáticos é difícil. Abordagens multidisciplinares - combinando modelagem ecológica, pesquisa molecular e vigilância epidemiológica - são essenciais para esclarecer as tendências impulsionadas pelo clima e melhorar o manejo das TBDs.

## Introduction

All living organisms are significantly affected by climatic factors, either directly (e.g., influencing their survival, growth, reproduction, and behavior) or indirectly (e.g., influencing habitat, food availability, and interaction) (Parmesan, 2006; Walther et al., 2002). Ticks are ectothermic, spend most of their life in the environment, and are particularly susceptible to climatic factors (Estrada-Peña et al., 2008; Randolph, 2008; Apanaskevich & Oliver, 2014). However, not all ticks are equally affected by climatic factors, with exophilic ticks being more influenced than nidicolous ticks and two/three-host ticks than one-host ticks (Sonenshine & Roe, 2014; Gray et al., 2009). Variations in temperature, precipitation, and humidity are key factors that influence ticks (Buczek et al., 2013). Although variations in other factors, including wind, photoperiod, solar radiation, clouds, and snowfall may influence ticks, these factors are yet to be fully investigated (Ostfeld & Brunner, 2015). Climate change-induced natural disasters such as floods, droughts, heat waves, wildfires, and ice melting can also affect ticks (Weiler et al., 2017; Berger et al., 2014; Bidder et al., 2019; Ogden et al., 2021). Extreme heat, drought, and flooding can reduce tick populations (via tick desiccation, drowning, or burial in silt, respectively) (Berger et al., 2014; Ogden et al., 2021; Weiler et al., 2017; Bidder et al., 2019). Wildfires may directly kill ticks (via killing hosts, disrupting habitats, and decreasing vegetation cover), while ice melt may prolong the tick activity period (MacDonald et al., 2018; Ogden et al., 2021).

**Figure 1:**
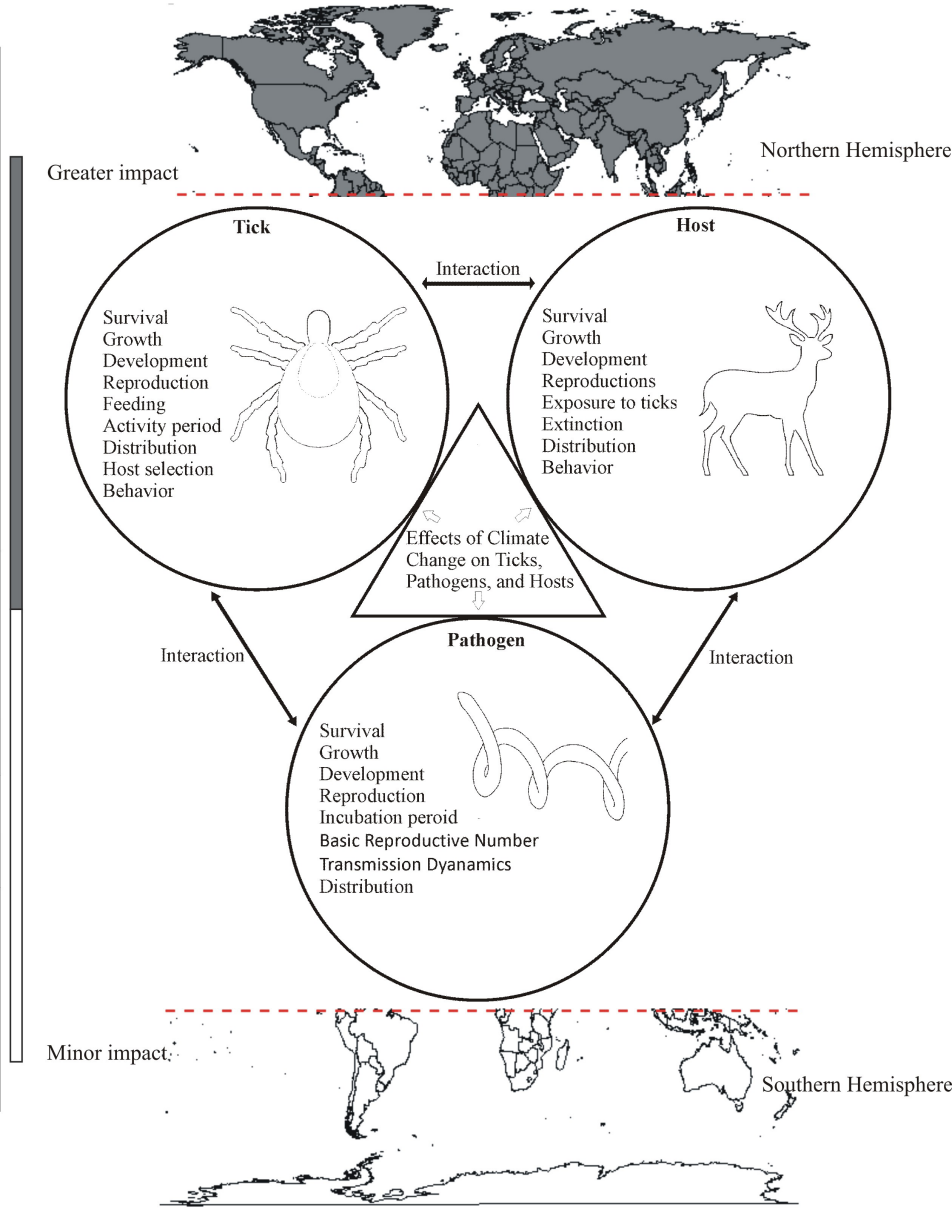
Schematic diagram showing how climate change (represented by triangle) can impact various aspects of ticks, hosts, and pathogens (represented by circles). The diagram also illustrates that the impacts in Northern Hemisphere are greater than Southern Hemisphere (represented by color gradient).

Global climate has changed over the last century, largely due to the emission of greenhouse gases from the continuous use of fossil fuels (Mikhaylov et al., 2020; Forster et al., 2024). Over global record (1850–2023), the ten warmest years have occurred in the last decade, with 2024 being the hottest year on record, at least so far (NASA Earth Observatory, 2024). In the next two decades, the global average surface temperature is expected to reach or exceed 1.5 °C, accompanied by other changes, including an increase in precipitation (IPCC, 2023). As a result, the biology and ecology of the tick-host-pathogen system have been significantly altered under climate change (Figure 1). Ticks may undergo changes in their lifecycle, behavior, and vector competency (Randolph & Rogers., 2000; Anderson et al., 2010; Gilbert et al., 2014; Ogden & Lindsay, 2016; Ginsberg et al., 2017; Nielebeck et al., 2023). Hosts may experience changes in population dynamics and behavior (Gilbert, 2010; Roy-Dufresne et al., 2013). Tick-borne pathogens may be influenced by shifts in transmission dynamics and vector ecology (Patz & Olson, 2006). The epidemiology of tick-borne diseases (TBDs), including shifts in geographical range, disease incidence, and host preference, has been significantly altered by these changes (Ogden et al., 2021; Medlock et al., 2013). While some of these changes have occurred in ways that have reduced tick-borne losses, most have occurred in ways that have increased them, which is the focus of this study (Randolph, 2009; Ogden & Lindsay, 2016). Although the impacts of climate crises on ticks and tick-borne pathogens (TTBPs) are worldwide, they are more prominent in the Northern Hemisphere, which is consistent with projected increases in temperature and precipitation in this region (Bouchard et al., 2019; Ogden et al., 2021). However, the current assessment is mostly based on limited measurable factors, and the actual effects may extend far beyond this (Ginsberg et al., 2017).

In addition to the 700,000 deaths per year from vector-borne diseases (VBDs), over 80% of the world’s population lives in areas at risk of at least one VBD (WHO, 2017, 2024). Although climate change is associated with the emergence and re-emergence of VBDs, which pose a serious risk to global health and the economy (Nuttall, 2022; Lee & Chung, 2023). Many aspects of TTBPs such as tick infestation on alternative hosts, have been neglected worldwide (Lee & Chung, 2023; Backus et al., 2021). However, some aspects, such as establishment in previously unaffected areas through host dispersal, including bird migration, have been studied in a limited number of species in Europe and North America (Dawe & Boutin, 2016; Sonenshine, 2018; Pascucci et al., 2019; Karim et al., 2024). Understanding these impacts is essential for mitigating the risks of TBDs to livestock, wildlife, and public health (WHO, 2024; Medlock et al., 2013).

As epidemiology of TBDs is also influenced by other anthropogenic drivers, including socioeconomic, demographic, land use changes (e.g., reforestation and suburbanization) (Stefanoff et al., 2012; Pfäffle et al., 2013; Bayles & Allan, 2014; Larsen et al., 2014), it is challenging to measure the actual effects of climate change. For instance, in Eastern United States (USA), the expansion of wooded areas in previously cleared landscapes, combined with increased human settlement at the wildland–urban interface, has facilitated greater contact between ticks and their hosts (Larsen et al., 2014). In addition to anthropogenic factors, recent advances in diagnostic technology (e.g., multiplex PCR and next-generation sequencing) have significantly influenced the reported incidence and diversity of TBDs (Michelet et al., 2014; Rowan et al., 2023). Nonetheless, certain approaches can help clarify climate-driven patterns, such as longitudinal studies conducted in ecosystems with limited human interference and advanced statistical methodologies. Examples of the former include the northward expansion of *I*. *persulcatus* and tick-borne encephalitis virus (TBEV) in northern Russia (Tokarevich et al., 2011), the range expansion of *Ixodes ricinus* in eastern Russia (Korotkov et al., 2015), the northward shift of *Ixodes scapularis* in southern Canada (Leighton et al., 2012), the altitudinal increase of TBEV in the Czech Republic (Kriz et al., 2012), and the rising incidence of TBEV infections in Sweden (Lindgren & Gustafson, 2001). These studies were carried out over long periods (20–30, 35, 25, 38, and 38 years, respectively) in areas where land use remain relatively. For the latter, examples comprise multivariate modeling techniques (e.g., generalized linear models, generalized additive models), species distribution models (e.g., MaxEnt, climate envelope models), and mechanistic models (e.g., population and transmission dynamics models (Gaff et al., 2020; Bregnard, 2021; Williams et al., 2025). This review encompasses previous studies mainly based on the following methodologies (a) field data (b) modeling approaches (c) statistical analyses, and (d) laboratory experiments (Parola et al., 2008; Leighton et al., 2012; Korotkov et al., 2015; Sage et al., 2017; Raghavan et al., 2019; Backus et al., 2021). Each of these approaches has its own strength and limitations, which needs to be recognized for guiding future research. For instance, ecological modeling has limitations, including non-equilibrium distributions, sampling bias, and variations in the spatial resolution of occurrence data (Alkishe et al., 2021). Additionally, controlled laboratory and mesocosm experiments, as well as comparative studies across landscapes, can also provide insights into climate-related changes (Stewart et al., 2013; Kilpatrick et al., 2017). In the context of climate change, this review focuses on the geographical expansion of TTBPs, the increase in the incidence of TBDs, and tick infestation on alternative hosts. Through a critical summary of previous knowledge, this review aims to identify potential challenges and opportunities for more effective control of ticks and TBDs in a changing climate.

## Climate Change Effects on Tick-Host-Pathogen Complex

Elevated temperature and humidity may enhance tick survival, growth, and reproduction, as demonstrated in both laboratory and field studies (Estrada-Peña et al., 2008; Ogden & Lindsay, 2016; Bouchard et al., 2019; Ogden et al., 2021). This may have partially contributed to an increase in the abundance of several tick species, (e.g., *Amblyomma americanum*, *Amblyomma maculatum*, *Dermacentor albipictus*, *Haemaphysalis longicornis*, *Hyalomma marginatum*, *Hyalomma rufipes*, *Ixodes cookie*, *I*. *ricinus*, *I*. *scapularis*, *Rhipicephalus microplus*, and *Rhipicephalus sanguineus*) (Tälleklint & Jaenson, 1998; Korotkov et al., 2015; Gray et al., 2009; Gasmi et al., 2019; Pouchet et al., 2024). Increases in temperature and humidity may have adjusted the seasonal synchrony among tick life stages, especially between the larval and nymphal stages, affecting the risk of transmission, as observed in studies on TBEV (Randolph & Rogers, 2000; Randolph, 2008), (Randolph & Rogers, 2000; Randolph, 2008). The co-feeding of infected and uninfected ticks of different species may also increase under warmer temperatures and increased humidity. Change in temperature and humidity may alter tick behavior in a non-linear way, with increased questing activity under moderate warming (Gilbert et al., 2014; Eisen et al., 2016; Ogden & Lindsay, 2016). As seen in southern populations of *I*. *scapularis*, climate change may lead to the evolution of exophilic ticks with nidicolous behavior, both increasing the chances of acquiring pathogens from hosts, which may serve as reservoirs for TBPs (Ginsberg et al., 2017). However, this shift can also reduce the efficiency of pathogen transmission cycles. Tick attachment rates to hosts may increase under elevated temperatures and lower humidity, as demonstrated in experimental studies with *R*. *sanguineus* (Socolovschi et al., 2009; Nielebeck et al., 2023). For instance, sea birds in Arctic Norway infested with *Ixodes uriae* experienced a 5% increase in infestation prevalence in the following breeding season, associated with an average 1 °C rise in winter temperatures (Descamps, 2013). In an experimental study, *R. sanguineus* ticks exposed to higher temperatures showed greater attachment to rabbits than ticks exposed to lower temperatures (Socolovschi et al., 2009). Global warming may have made ticks more aggressive, causing them to bite atypical hosts (Parola et al., 2008; Backus et al., 2021). The vector competency of ticks may have been influenced by increasing temperatures, a phenomenon well studied in mosquitoes, though direct evidence for ticks is less robust and needs further research (Anderson et al., 2010; Westbrook et al., 2010). The dynamics of acaricides, such as effectiveness and resistance, can be affected by climate change owing to a potential increase in the amount and duration of acaricide treatment.

Variations in precipitation patterns and rising temperatures may also have increased the abundance and altered the migration patterns of TTBPs hosts (Childs & Paddock, 2003; Peh, 2007; Cagnacci et al., 2011; Jaenson & Lindgren, 2011; Jaenson et al., 2012; Roy-Dufresne et al., 2013; Dawe & Boutin, 2016; Wikel, 2018; Bouchard et al., 2019). Compared to domestic animals, wild animals may play a more significant role as they are reservoirs of TBPs and less influenced by human interventions. Increases in abundance and shifts in migration patterns have been observed in various TTBPs hosts (e.g., birds, bats, deer, dogs, hares, pigs, and rodents) (Childs & Paddock, 2003; Peh, 2007; Cagnacci et al., 2011; Jaenson & Lindgren, 2011; Jaenson et al., 2012; Roy-Dufresne et al., 2013; Dawe & Boutin, 2016; Wikel, 2018; Bouchard et al., 2019). For example, the population size and geographical distribution of white-tailed deer (*Odocoileus virginianus*) and white-footed mice (*Peromyscus leucopus*) in North America and roe deer (*Capreolus capreolus*) in Europe have increased (Childs & Paddock, 2003; Cagnacci et al., 2011; Jaenson & Lindgren, 2011; Jaenson et al., 2012; Roy-Dufresne et al., 2013; Dawe & Boutin, 2016; Wikel, 2018). Shifts in population size and geographical distribution in response to climate variability have been studied in several hosts (e.g., birds and bats), however, these findings have not yet been widely translated to the TTBPs associated with these hosts (Peh, 2007; Diengdoh et al., 2022). The decline in certain host species owing to climate change could lead to an increase in the number of generalist species that are more adaptable to environmental changes. For instance, the abundance of rodents, which mainly serve as hosts for nymphs (the main vectors of certain pathogens), has increased owing to the decrease in its predator, the red fox population (Levi et al., 2012). Examples include *P*. *leucopus* associated with *I*. *scapularis* ticks and *Borrelia burgdorferi* and *Anaplasma phagocytophilum* pathogens in North America and bank vole (*Clethrionomys glareolus*), yellow-necked mouse (*Apodemus flavicollis*), and *Microtus arvalis* associated with *I*. *ricinus* ticks and *B*. *burgdorferi* and TBEV (Levi et al., 2012; Tkadlec et al., 2019; Beermann et al., 2023). This decline is linked to the expansion of the coyote population, which expanded following the decline in gray wolves (Gompper, 2002; Levi et al., 2012). It is not fully understood, however, the impacts of climate change on the dynamics of these predators cannot be ignored. Animals, either independently or through their owners, may migrate to search for favorable conditions. The mixing of wildlife and livestock, due to their movement, may occur in the search for a suitable environment. For instance, during the 2009 drought in West Kilimanjaro, Tanzania, elephants left protected areas and entered villages and farmlands, where they encountered cattle, goats, and sheep (Mariki et al., 2015). Climate change-induced stress may affect host immunity either directly through physiological changes or indirectly through other factors (e.g., food, infections, and parasitism); however, this aspect has received less attention. Moreover, changes in host behavior may occur. For example, humans may spend more time outdoors, have a greater proportion of their bodies exposed, and inhabit areas closer to forests and other vegetation. Climate change can facilitate host interactions such as the sharing of breeding sites between different host species. The seasonal synchrony between ticks and their hosts may be enhanced by climate change.

Rising temperatures can affect TBPs replication rate, basic reproductive number, and extrinsic incubation period of mosquito-associated pathogens (Ochanda et al., 1988; Dohm et al., 2002; Patz & Olson, 2006; Anderson et al., 2010; Daniel et al., 2018). However, the direct impacts of climate change on TBPs have been neglected. Climate change may promote other aspects of the TBPs, such as the number of vectors, spillovers, infection rates, and co-infection with pathogens.

**Figure 2:**
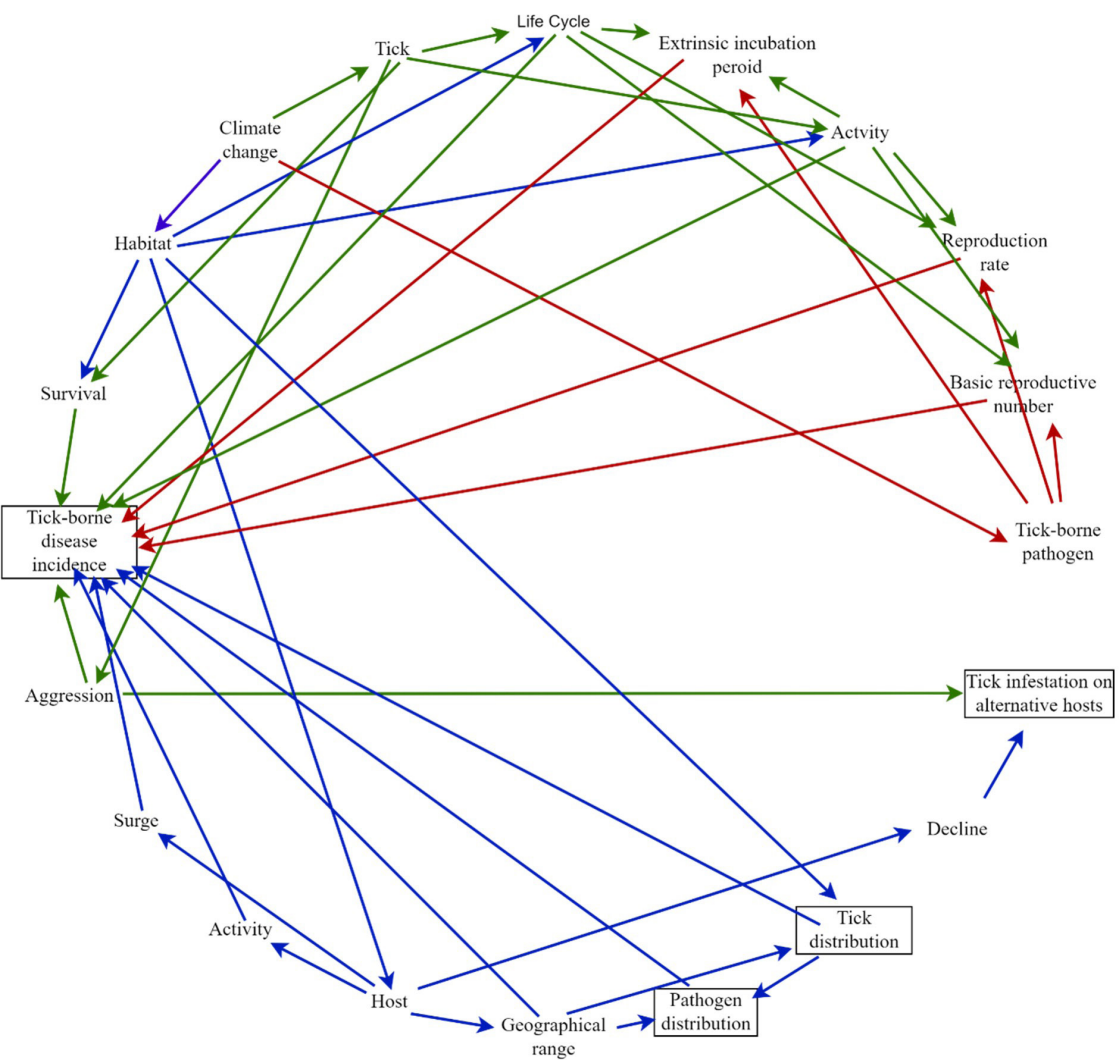
Impact of climate change on epidemiology of ticks and tick-borne diseases (ticks and pathogens distribution, tick-borne disease incidence, and tick infestation on alternative hosts) through ticks (green arrows), tick-borne pathogens (red arrows) and habitat (blue arrows).

These changes in tick, host, and pathogen dynamics due to climate change may ultimately affect tick distribution, pathogen distribution, incidence of tick-borne diseases, and tick infestation in alternative hosts (Figure 2). However, attributing these changes solely to climate is challenging, since many of these are also influenced by other factors, including landscape modifications (Childs & Paddock, 2003; Stefanoff et al., 2012; Larsen et al., 2014; Gilbert, 2021).

## Impact of Climate Change on Tick Distribution

Climate change could have partially influenced tick distribution by affecting ticks, their hosts, and the habitats of both (Estrada-Peña et al., 2008; Cagnacci et al., 2011; Jaenson & Lindgren, 2011; Jaenson et al., 2012; Roy-Dufresne et al., 2013; Dawe & Boutin, 2016; Ogden & Lindsay, 2016; Bouchard et al., 2019; Ogden et al., 2021). *Haemaphysalis longicornis*, native to Eastern Asia, has expanded into the United States (Tufts & Diuk-Wasser, 2021). The distribution of other tick species, such as *Ixodes cookei*, *I*. *scapularis*, *A*. *maculatum*, and *A*. *americanum*, has expanded in the United States and Canada (Teel et al., 2010; Leighton et al., 2012; Feria-Arroyo et al., 2014; Florin et al., 2014; Springer et al., 2015; Monzón et al., 2016; Scott & Scott, 2018; Sonenshine, 2018; Cuervo et al., 2021). Tick species, such as *Dermacentor andersoni*, are expected to extend their range in North America (Alkishe & Peterson, 2022). Other ticks, such as *Hyalomma marginatum* and *Hyalomma Rufipes* have been reported to spread from Africa to Europe, including Hungary, England, France, Italy, Slovenia, Germany, and Sweden (Trilar, 2004; Rumer et al., 2011; Hornok & Horváth, 2012; Jameson et al., 2012; Toma et al., 2014; Vial et al., 2016; Pascucci et al., 2019; Hansford et al., 2019; McGinley et al., 2021). *Dermacentor reticulatus* has extended its range to Germany and the Netherlands (Dautel et al., 2006; Nijhof et al., 2007; Karbowiak & Kiewra, 2010; Paulauskas et al., 2015; Drehmann et al., 2020), the Czech Republic, Baltic countries (Lithuania and Latvia), and Poland. In addition to the increase in the geographical distribution of *I. ricinus* in Finland, the expansion of this species from plains to mountainous regions has been documented in Sweden, Norway, Spain, Italy, Greece, Portugal, France, the Balkans, and the Czech Republic (Lindgren et al., 2000; Daniel et al., 2003; Materna et al., 2005; Gray et al., 2009; Jore et al., 2011; Jaenson et al., 2012; Medlock et al., 2013; Li et al., 2019). In Sweden and Finland, *I*. *persulcatus* has expanded to European Russia (Bugmyrin et al., 2013; Jaenson et al., 2016; Laaksonen et al., 2017). *Amblyomma variegatum* and *Amblyomma hebraeum* have been reported to have expanded their range further in Zimbabwe (Estrada-Peña et al., 2008). It has been reported that some tick species have to spread while replacing other tick species. For example, *Rhipicephalus decoloratus* and *Rhipicephalus geigyi* are being superseded by *R.microplus* in African countries, including South Africa, Zimbabwe, Ivory Coast and Tanzania (Tønnesen et al., 2004; Lynen et al., 2008; Madder et al., 2007, 2011).

Ecological modeling predicts that climate change may further extend tick distribution. For instance, in addition to the current expansion of *I*. *cookei*, *I*. *scapularis* and *A*. *americanum* in the USA and Canada, the distribution of *Ixodes pacificus*, *Dermacentor variabilis*, *D*. *andersoni* and *R*. *sanguineus sensu lato* is expected to expand in Canada. Similarly, *D*. *variabilis* and *Ornithodoros hermsi* are expected to expand in the USA (Sage et al., 2017; Raghavan et al., 2019; Boorgula et al., 2020; Fish, 2021; Nuttall, 2022; Sonenshine, 2018). In the USA, the reintroduction and re-establishment of *R*. *microplus* are anticipated (Giles et al., 2014). *Hyalomma marginatum* may potentially spread to western Palearctic regions, including Europe, whereas *Hyalomma dromedarii* may extend its distribution to North Africa (Salem et al., 2011; Estrada-Peña et al., 2012). *Rhipicephalus sanguineus*, currently limited to Mediterranean regions in Europe, may shift further northwards (Gray et al., 2009), while *D*. *reticulatus* is expected to expand its range in Europe (Zając et al., 2021). Habitat expansion has been predicted for several tick species. For example, *I*. *ricinus* habitats are expected to expand by 3.8% in Europe, affecting countries such as Belarus, Denmark, Estonia, Finland, Latvia, Lithuania, Norway, and Sweden during 2020–2040 (Boeckmann & Joyner, 2014). In Columbia, the habitat expansion of *Amblyomma ovale* and *Amblyomma maculatum* has been predicted for 2070, while decrease for *Amblyomma mixtum* and *Amblyomma patinoi* is expected in near future in Caribbean, Andean and Pacific region (Polo et al., 2024). In China, the habitat expansion of *Hyalomma asiaticum*, *Rhipicephalus turanicus*, *Dermacentor marginatus*, and *Haemaphysalis punctata* is expected (Ma et al., 2024). Conversely, the distribution range of some tick species is expected to decrease, such as *I*. *cookei*, and *A*. *americanum* in the southern USA (Alkishe et al., 2021), *I*. *ricinus* in parts of Europe (Croatia, France, Germany, Italy, and Spain) (Boeckmann & Joyner, 2014). Similarly, the range of several other tick species is expected to shrink. In South Africa, this includes *A*. *hebraeum*, *Hyalomma truncatum*, *Rhipicephalus appendiculatus*, and *R*. *decoloratus* (Estrada-Peña, 2003), while in Brazil, *Amblyomma cajennense* (sensu stricto) and *Amblyomma sculptum*, and in Anatolia, *Hyalomma marginatum* are also expected to decline (Hekimoglu et al., 2023). By 2050 and 2070, the areas suitable for *R. microplus* are expected to expand worldwide, particularly in regions with large cattle populations (Marques et al., 2020). Predicted for 2050, habitat suitability for *R. sanguineus* is increase in the western United States, Venezuela, Uruguay, Brazil and Bolivia, while decrease in Midwest and southern United State, Peru, Guyana and Argentina. By 2070, further expansion of *R. sanguineus* is expected in the western United State, Brazil and Bolivia, while declining in northern Brazil, southern United State, Paraguay, and central Argentina (Sánchez Pérez et al., 2023). These shifts not only introduce ticks to previously unaffected regions but also elevate the risk of TBDs in these areas.

## Impact of Climate Change on Tick-Borne Pathogen Distribution

Climate change has influenced TBPs distribution by affecting TBPs themselves, as well as their tick and vertebrate hosts (Estrada-Peña et al., 2008; Cagnacci et al., 2011; Jaenson & Lindgren, 2011; Jaenson et al., 2012; Roy-Dufresne et al., 2013; Dawe & Boutin, 2016; Ogden & Lindsay, 2016; Wikel, 2018; Bouchard et al., 2019; Ogden et al., 2021; Daniel et al., 2018). *Borrelia burgdorferi*, *A*. *phagocytophilum* (associated with *I. scapularis*) and *Rickettsia rickettsii* (associated with *R*. *sanguineus*) are thought to have expanded their geographical ranges in the USA (Hamer et al., 2010; Wikel, 2018). Various TBPs, including *B. burgdorferi*, *A. phagocytophilum, Babesia microti,* Powassan virus, *Borrelia miyamotoi* (associated with *I. scapularis*), and Powassan virus (associated with *I. cookei*) have emerged in Canada (Bullard et al., 2014; Dibernardo et al., 2014; Krakowetz et al., 2014; O’Brien et al., 2016; Corrin et al., 2018; Gasmi et al., 2017; Edginton et al., 2018). In Brazil, the increased risk and spread of *Anaplasma marginale* and *Babesia* spp., which are associated with *R. microplus*, has been examined (Puentes & Riet-Correa, 2023). In addition to the increased risk of *B. burgdorferi* and TBEV (associated with *I. ricinus*) in Sweden and Slovenia, their expansion to higher altitudes has also been reported in the Czech Republic (Gustafson et al., 1995; Daniel et al., 2004; Danielová et al., 2006; Lindgren & Gustafson, 2001; Zeman & Beneš, 2004; Donša et al., 2021). Tick-borne encephalitis virus, which is associated with *I. scapularis*, has also spread to European Russia, Switzerland, and Austria (Gäumann et al., 2010; Tokarevich et al., 2011; Heinz et al., 2015). New occurrences of *Babesia canis*, linked to *D*. *reticulatus*, have been reported in Germany, Switzerland, the Netherlands, and Hungary (Sréter et al., 2005; Heile et al., 2006; Nijhof et al., 2007; Porchet et al., 2007; Barutzki et al., 2007). Recently, the Crimean-Congo hemorrhagic fever virus (associated with *Hyalomma* spp.) was detected in *Hy. marginatum* in France (Bernard et al., 2024). The increase distribution of *Ehrlichia hydrochoerus* (associated with *A. dubitatum*) in Argentina was found to be positively correlated with elevated minimum temperatures and elevated cumulative rainfall (Eberhardt et al., 2023). The geographic range of *Borrelia crocidurae* (associated with *Ornithodoros* ticks) extends from the Sahelo-Saharan region of Africa into northwestern to Morocco (Souidi et al., 2014). Therefore, climate change may continue to increase distribution of TBPs. For instance, *Anaplasma marginale* (associated with *Dermacentor* spp.), *Ehrlichia chaffeensis* (associated with *A*. *americanum*), *R*. *rickettsii* (associated with *Dermacentor* spp. and *R. sanguineus*), and *Borrelia hermsii* (associated with *Ornithodoros hermsi*) are expected to spread with the expansion of their vectors in North America (Alkishe et al., 2021). If climate change allows cattle egrets to spread into the US from the southern regions, it may disseminate the infected *A*. *variegatum*. Even without the establishment of this tick species, its pathogen, *Ehrlichia ruminantium*, could still spread and be maintained because of the presence of the native vector *A*. *maculatum* and the vertebrate reservoir deer. Similarly, the CCHF virus may have spread to the western Palearctic regions. Moreover, climate change is linked to the emergence of several TBPs, such as the Yezo virus in China and Japan (possibly associated with *I*. *persulcatus*) and the thrombocytopenia syndrome virus in Japan (potentially associated with *Haemaphysalis* spp.) (Kumar et al., 2024; Wagatsuma, 2024). The UK Health Security Agency (UKHSA) concluded that climate change could expand the distribution of several tick species in the UK that are of public health concern, including *I. ricinus*, a vector for the agents that cause Lyme disease and tick-borne encephalitis. Additionally, the expansion of *D. reticulatus* and *Haemaphysalis punctata* was observed, both of which are known to occasionally bite humans. As the climate continues to warm and the presence of these parasites persists, the risk of tick-borne disease transmission in the UK is expected to increase (UK Health Security Agency, 2023). In addition to facilitating the emergence and re-emergence of TBPs in new regions, the distribution of TBPs under climate change conditions also increases the incidence of TBD. Moreover, tick-borne pathogens can help ticks cope with harsh environmental conditions. For example, *A*. *phagocytophilum* infection triggers the production of stress proteins in ticks (Neelakanta et al., 2010), while *Borrelia afzelii* infection appears to improve tick survival in dry conditions, possibly by increasing fat storage (Herrmann et al., 2013). Climate change may influence the microbial communities within ticks, though research on this topic is still very limited, and the specific mechanisms involved remain largely unclear. Changes in microbiota could potentially enhance tick stress tolerance, alter their behavior, help them adapt to new environments, and even impact the transmission of tick-borne diseases (Thapa et al., 2019; Cabezas-Cruz, 2021).

## Impact of Climate Change on Tick-Borne Disease Incidence

Climate change might affect the incidence of TBDs by affecting ticks, their associated pathogens, and vertebrate hosts (Randolph & Rogers, 2000; Estrada-Peña et al., 2008; Randolph, 2008; Cagnacci et al., 2011; Jaenson & Lindgren, 2011; Jaenson et al., 2012; Levi et al., 2012; Roy-Dufresne et al., 2013; Dawe & Boutin, 2016; Ogden & Lindsay, 2016; Wikel, 2018; Bouchard et al., 2019; Ogden et al., 2021; Daniel et al., 2018; Backus et al., 2021; Nielebeck et al., 2023). In the USA, the incidence of LD (caused by *B*. *burgdorferi*) (s.l.) has approximately doubled, from 3.74 reported cases in 1991 to 7.21 reported cases per 100,000 people in 2018 (Dumic & Severnini, 2018). From 2007 to 2021, Brazil reported over 36,500 suspected cases of Brazilian Spotted Fever (BSF) (caused by *Rickettsia rickettsii* associated with *Amblyomma sculptum*), with 7% confirmed, averaging 170 cases and causing 837 deaths over 15 years (Brasil, 2025). The incidence of anaplasmosis (caused by *A*. *phagocytophilum*) increased from 1.4 cases per 1,000,000 persons in 2000 to 8.0 cases in 2012 (Dahlgren et al., 2015). Similarly, during 2000–2012, the incidence of spotted fever rickettsioses (caused by rickettsial agents, e.g., *R*. *rickettsii*, *Rickettsia parkeri*, and other *Rickettsia* spp.) increased from 1.7 to 14.3 cases per million persons (Openshaw et al., 2010; Drexler et al., 2016). In Canada, between 2011–2021, the incidence of Lyme disease has increased from 0.8 cases to 8.2 cases per 100,000 inhabitants (Government of Canada, Surveillance of LD). In Europe, the incidence of LD has increased in several countries including the Czech Republic, Estonia, Sweden, Finland, Germany, and Poland (ECDC, 2014; Stefanoff et al., 2014; Enkelmann et al., 2018; Li et al., 2019; Burn et al., 2023). For instance, between 1999 and 2008, the Czech Republic experienced an increase in the LD incidence from 23.6 to 46.5 cases per 100,000 people, whereas an increase in the LD incidence from 1.0 to 25.2 cases per 100,000 inhabitants was noted in Poland during the same period (Stefanoff et al., 2014). With an estimated 400% increase in the prevalence of TBE in endemic regions during three decades till 2014, increased incidence of TBE has documented in various European countries, such as Czech Republic, Norway, and Sweden, as well as in European Russia (Lindgren & Gustafson, 2001; ECDC, 2014; Kříž et al., 2018; Li et al., 2019; Tokarevich et al., 2011). The incidence of SFR (caused by rickettsial agents such as *Rickettsia conorii* and *Rickettsia massiliae* associated with *R. sanguineus*) has increased in European countries, including Italy and France (Parola et al., 2008; Vescio et al., 2008). Similarly, human cases of the Powassan virus are increasing across its range, which can be attributed to climate change. The incidence of Crimean-Congo hemorrhagic fever (CCHF) is also increasing in countries such as Turkey and Iran (Ansari et al., 2014; Ahmadkhani et al., 2018; Duygu et al., 2018). Moreover, the increasing number of CCHF cases in Pakistan, potentially owing to climate change, requires further investigation (Abid et al., 2025). The incidence of CCHF may increase in the future; for instance, by 2050, the projected annual number of LD cases in Canada is expected to be between 120,000 and 500,000 (Ogden et al., 2024). In Slovenia, the LD risk is expected to increase by 10% by the end of the century (Donša et al., 2021). In contrast, a decrease in LD cases is expected in some parts of the world, including southern Europe (Cox et al., 2021). Additionally, in Ireland, alterations have been observed in the development and activity of *Ixodes ricinus* throughout the year, which are likely due to increased global warming. These changes may also be linked to shifts in the seasonal patterns of tick-borne diseases (Gray, 2008). More recently, two studies using different modeling approaches analyzed the potential risk of increasing tick populations associated with environmental and climate changes (Cao et al., 2025; Teo et al., 2024). Observed changes in TBD incidence may pose potential health and economic consequences, with climate change being one of several interacting drivers.

## Impact of Climate Change on Tick Infestation on Alternative Hosts

Previously, tick infestation on alternative hosts has been attributed to heavy infestation of usual hosts and the environment. An example is *R. sanguineus* and its primary dog host (Dantas-Torres et al., 2006). Recently, some studies have revealed the role of high temperatures in host preference of this tick species (Backus et al., 2021). Over the last 50 years, an increase in SFR cases, including Mediterranean spotted fever, has been recorded in countries bordering the Mediterranean region, including Spain, France, Italy (from Europe), and Algeria (Africa) (Arenas et al., 1986; Mansuelo et al., 1986; Gilot et al., 1990; Raoult et al., 1992; Parola et al., 2005; Mouffok et al., 2006; Parola et al., 2008; Vescio et al., 2008). Notably, these increases occur during the warmest years in these regions. Such situations correlate with the increased aggressiveness of *R. sanguineus*, which infests humans more frequently with rising temperatures. In some cases, dense populations of *R*. *sanguineus* were found on the exact hosts infected with MSF or other hosts and in the environment. In Marseille, France, in 2003, a homeless alcoholic man who died of MSF was heavily infested with 22 *R*. *sanguineus* ticks (Parola et al., 2005). The preference of *R*. *sanguineus* for infesting humans at high temperatures has been experimentally validated (Parola et al., 2008). Other studies, such as that of Backus et al. (2021), have experimentally validated that *R. sanguineus*, particularly the tropical lineage, prefers humans to dogs at high temperatures. Many other cases of tick infestation on alternative hosts occur amid ongoing climate change, such as *R*. *microplus* infestation of goats and wildlife in African countries, including Benin and South Africa (Nyangiwe & Horak, 2007; Tonetti et al., 2009; Adinci et al., 2018), which are yet to be evaluated for the involvement of climate change. Changes in tick-host interactions under climate change not only affect native hosts but also exert pressure on naïve hosts via tick infestation, opening new avenues for research. Although there’s limited research directly linking climate change to shifts in host immunity, climate extremes like heatwaves and drought can weaken the immune system, making both typical and alternative hosts more vulnerable to tick infestations (Bagath et al., 2019; Ogden et al., 2021).

## Underlying Mechanism

Although climate change affects the epidemiology of TTBPs by influencing several aspects (Figure 2), there is limited information on the underlying biological mechanisms. Understanding these mechanisms is crucial for predicting future tick-borne disease dynamics and developing mitigation strategies. Climate change can impact the tick life cycle (via altering metabolic rates, which in turn may affect the pre-oviposition period, egg development, interstadial development, and developmental diapause), activity (via changes in cuticle permeability, thermal thresholds, behavioral diapause, and periods of quiescence), and aggression (via energy depletion, sensitivity to human chemical cues, and shifts in microhabitat) (Beament, 1959; Ogden et al., 2004; Belozerov, 2009; Cabrera & Labruna, 2009; Estrada-Peña et al., 2012; Burtis et al., 2019). Physiological responses, such as the expression of heat shock proteins and temperature-sensitive genes, also contribute to tick resilience under environmental stress (Villar et al., 2010; Busby et al., 2012). Climate change also affects the basic reproductive number of TTBPs (via influencing the tick life cycle and seasonal synchrony, host activity and migration, and increased host-tick encounters), reproduction rate (via metabolic changes), and extrinsic incubation period (via physiological development) (Ogden et al., 2014). Moreover, temperature-dependent gene regulation in pathogens, affecting its colonization, transmission, and survival (Couret et al., 2022). Moreover, climate change affects habitat (via altering vegetation, shelter availability, soil conditions, and overall environmental suitability, host availability) (Gray et al., 2009; Jaenson & Lindgren, 2011).

## Knowledge Gaps

Overall, the shift in the epidemiology of TBPs under ongoing climate change is occurring across more aspects, involving more species, and over a wider area than they are being studied in relation to climate change. Many aspects, such as tick infestation on alternative hosts and genetic mutations in ticks, have been largely neglected, and among the studied aspects, such as TTBPs distribution and TBDs incidence, research has been focused on limited species and areas. Although the impact of climate change on TBPs distribution and TBDs incidence via ticks has been studied, the direct effects are poorly understood. Evidence of the impacts of climate change on TBPs epidemiology mostly comes from cross-sectional studies, which capture only specific points in time, while longitudinal studies that show trends over longer time periods are largely missing. The biological mechanisms behind these changes are still not well understood. Limited data and simplified models have sometimes led to unreliable predictions. Improving our knowledge in these areas is essential for developing effective strategies to manage TBDs under climate change.

## Concluding Remarks

In parallel with climate change, the epidemiology of TTBPs has changed, characterized by an expansion in their geographical range, increased incidence of TBDs, and tick infestation on alternative hosts. Yet, the actual impacts of climate change on the epidemiology of TBDs remains unclear. Multidisciplinary approaches, such as integrating ecological modeling, molecular research, and epidemiological surveillance may help understand these effects. Policymakers should focus on implementing comprehensive strategies, such as enhanced disease surveillance and public health preparedness to mitigate associated health risks. Although evidence suggest that climate change influences the epidemiology of TBDs, its effects cannot be considered in isolation. Other factors such as variations in host population, land use, and advances in diagnostic approaches also shape the distribution patterns of TBDs. Additionally, separating these overlapping factors remains a key challenge, and future research should focus on clarifying their relative contributions.

## Data Availability

No data was used for the research described in the article.
